# NURSING HOME WORKFORCE EDUCATION: DOES ONE SIZE FIT ALL? EVIDENCE FROM THE CHATO NATIONAL TRIAL

**DOI:** 10.1093/geroni/igad104.2413

**Published:** 2023-12-21

**Authors:** Kristine Williams, Yelena Perkhounkova, Carissa Coleman, Maria Hein, Frances Yang, Clarissa Shaw

**Affiliations:** University of Kansas Medical Center, Kansas City, Kansas, United States; University of Iowa, Iowa City, Iowa, United States; University of Kansas, Kansas City, Kansas, United States; University of Iowa, College of Nursing, Iowa City, Iowa, United States; University of Kansas, Kansas City, Kansas, United States; University of Iowa, Iowa City, Iowa, United States

## Abstract

Educating the nursing home workforce to provide quality dementia care is a national priority. Staff education is usually provided to all staff in communities that vary in size, location, ownership, and resources with little consideration for diversity. To evaluate suitability for diverse staff, we compared learning outcomes for White, non-Hispanic staff and staff of diverse races and ethnicities who completed CHATO in year one. White non-Hispanic staff (N=402) scored higher on knowledge assessments than staff self-reporting other races and ethnicities (N=201) at baseline (mean=57.1% vs. 49.1%, p<.001). Both groups improved on post-assessment (by 19.0 and 23.1 percentage points, respectively, p<.001), Communication ratings indicated that at baseline White, non-Hispanic staff were more knowledgeable about effective (p<.001), appropriate (p=.008), and person-centered communication (p<.001) than diverse staff; while the latter recognized elderspeak better (p=.002). Both groups improved their communication ratings post-CHATO (p from .001 to <.001). While confidence in providing dementia care was similar between groups at baseline (p=.754), it improved only for White, non-Hispanic staff (p<.001). Intention to use learned skills in practice was higher for staff in the diverse group (p<.001). Diverse staff rated their satisfaction with the intervention lower (p <.001), suggesting that it was not tailored to their needs. These findings support the value of CHATO in increasing communication knowledge among diverse NH staff. However, lower satisfaction suggests the need to adapt CHATO to be more inclusive and culturally appropriate. Tailoring education may increase impact on outcomes and overcome disparities in dementia care common in NHs serving diverse populations.

